# Hydrogen peroxide is involved in hydrogen sulfide-induced lateral root formation in tomato seedlings

**DOI:** 10.1186/s12870-017-1110-7

**Published:** 2017-10-13

**Authors:** Yudong Mei, Haotian Chen, Wenbiao Shen, Wei Shen, Liqin Huang

**Affiliations:** 1 College of Life Sciences, Laboratory Center of Life Sciences, Nanjing Agricultural University, Nanjing, 210095 China; 2 College of Sciences, Nanjing Agricultural University, Nanjing, 210095 China

**Keywords:** Hydrogen sulfide (H_2_S), Hydrogen peroxide (H_2_O_2_), *Solanum lycopersicum*, Lateral root formation, miRNA, *S*-sulfhydration

## Abstract

**Background:**

Both hydrogen sulfide (H_2_S) and hydrogen peroxide (H_2_O_2_) are separately regarded as a highly reactive molecule involved in root morphogenesis. In this report, corresponding causal link governing lateral root formation was investigated.

**Methods:**

By using pharmacological, anatomic, and molecular approaches, evidence presented here revealed the molecular mechanism underlying tomato lateral root development triggered by H_2_S.

**Results:**

A H_2_S donor sodium hydrosulfide (NaHS) triggered the accumulation of H_2_O_2_, the up-regulation of *RBOH1* transcript, and thereafter tomato lateral root formation. Above responses were sensitive to the H_2_O_2_ scavenger (dimethylthiourea; DMTU) and the inhibitor of NADPH oxidase (diphenylene idonium; DPI), showing that the accumulations of H_2_O_2_ and increased *RBOH1* transcript were respectively prevented. Lateral root primordial and lateral root formation were also impaired. Further molecular evidence revealed that H_2_S-modulated gene expression of cell cycle regulatory genes, including up-regulation of *SlCYCA2;1*, *SlCYCA3;1*, and *SlCDKA1*, and the down-regulation of *SlKRP2*, were prevented by the co-treatment with DMTU or DPI. Above mentioned inducing phenotypes were consistent with the changes of lateral root formation-related microRNA transcripts: up-regulation of *miR390a* and *miR160*, and with the opposite tendencies of their target genes (encoding auxin response factors). Contrasting tendencies were observed when DMTU or DPI was added together. The occurrence of H_2_S*-*mediated *S*-sulfhydration during above responses was preliminarily discovered.

**Conclusions:**

Overall, these results suggested an important role of *RBOH1*-mediated H_2_O_2_ in H_2_S-elicited tomato lateral root development, and corresponding H_2_S-target proteins regulated at transcriptional and post-translational levels.

**Electronic supplementary material:**

The online version of this article (10.1186/s12870-017-1110-7) contains supplementary material, which is available to authorized users.

## Background

Lateral root (LR) formation, which entirely originated from pericycle founder cells, is of critical importance for the plant root architecture [1]. Normally, LR formation depends on both genetic determinants and postembryonic developmental processes that are mainly under the influence of plant hormone (usually auxin) and environmental factors, including water and nutrient availability [1, 2]. Genetic and molecular evidence revealed that auxin regulates LR formation by modulating the transcripts of cell cycle regulatory genes, such as cyclins and Cyclin Dependent Kinases (CDK) in the pericycle cells [3–6]. Previous results showed that nitric oxide (NO) mediated the activation of auxin-dependent cell cycle regulatory genes encoding CYCA2;1, CYCA3;1, CDKA1, and the cell cycle inhibitor Kip-Related Protein KRP2 in tomato seedlings at the beginning of LR primordia formation [6]. On the other hand, auxin response factors (ARFs) appeared to play an essential role in auxin-regulated gene expression during plant development, including LR formation, etc. [7–9]. A decade ago, a class of small, non-coding RNAs, called microRNAs (miRNAs), was identified to regulate gene expression [10, 11]. Several miRNAs related to ARFs have been detected via computational approaches [12], such as *miR390* targeting *ARF2*, *ARF3* and *ARF4* [13], while *miR160* targeting *ARF10*, *ARF16* and *ARF17* [14].

After NO and carbon monoxide (CO) [15], hydrogen sulfide (H_2_S) is proposed as the third gaseous messenger to be involved in guard cell signaling [16], root organogenesis [17], and the alleviation of seed germination inhibition caused by heavy metal exposure [18]. In mammalian cells, H_2_S can be endogenously generated from four enzymes, such as cystathionine-*γ*-lyase (CSE), cystathionine-*β*-synthase (CBS), cysteine aminotransferase, and 3-mercaptopyruvate sulfurtransferase (3-MST) [19, 20]. In plants, H_2_S synthesis is partially catalyzed by _L_-cysteine desulfhydrase (DES; homolog with CSE in animals) [21, 22]. Related experiments discovered that H_2_S might be involved in auxin-induced LR formation in tomato seedlings [23]. Importantly, the discovered mechanism of physiological effects achieved by H_2_S in animals and recently in plants is *S*-sulfhydration: a posttranslational modification of protein cysteine residues (persulfide R-SSH formation) [24–26]. Above modification manner is opposed to *S*-nitrosylation, another posttranslational modification of protein cysteine residues by NO with the formation of *S*-nitrosocysteine residues (R-SNO) [27]. However, whether protein *S*-sulfhydration was involved in H_2_S-mediated LR formation, is still unknown.

It was well-known that hydrogen peroxide (H_2_O_2_) plays various vital roles in signal transduction beside its toxic effects. In fact, H_2_O_2_ is an important product of NADPH oxidase, polyamine oxidases (PAO), and diamine oxidases (DAO), etc. [28, 29]. Subsequent results showed that H_2_O_2_ mediates plant responses against adversity stresses and takes part in plant development processes, including stomatal closure [30], root gravitropism [31], and cell elongation [32]. Specially, H_2_O_2_ is also involved in auxin signaling [31, 33, 34], adventitious rooting [34, 35], and LR formation [36–39].

Although H_2_S and H_2_O_2_ were respectively suggested to be required for root architecture [17, 32], the cross-talk between H_2_S and H_2_O_2_ in tomato LR development, has not been fully elucidated. In this report, the analysis of H_2_S-regulated mechanisms leading to LR promotion is expanded. By using pharmacological, anatomic, and molecular approaches, evidence presented here supported the role of *RBOH1*-mediated H_2_O_2_ in the regulation of tomato LR development achieved by H_2_S. Potential mechanisms, including LR-related ARFs gene expression via miRNAs, are preliminarily elucidated. Additionally, downstream signaling events modulated by H_2_S might occur in both transcriptional and posttranslational levels (protein *S*-sulfhydration, etc.). Above results thus provide insights into H_2_S signaling in plant development.

## Results

### Increases of endogenous **H**_**2**_**O**_**2**_ contents and **LR** formation elicited by NaHS

**Fig. 1:**
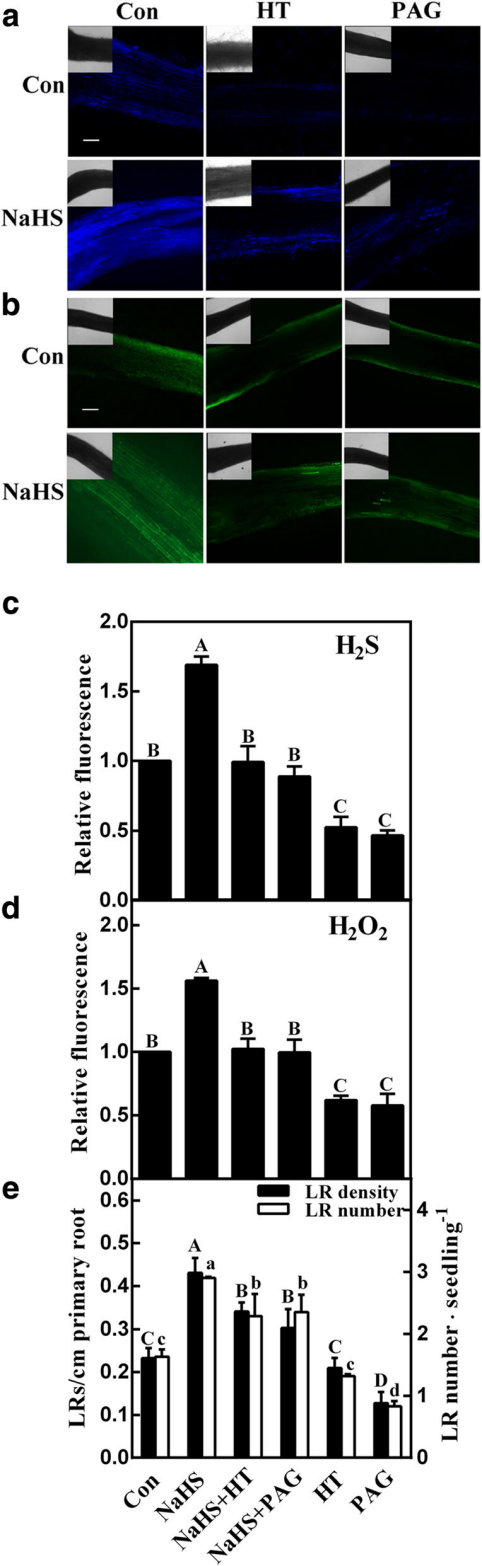
Sodium hydrosulfide (NaHS; the H_2_S donor) increases H_2_O_2_ accumulation and thereafter lateral root (LR) formation. Three-day-old tomato seedlings were treated with H_2_O (Con), 1 mM NaHS, 200 μΜ hypotaurine (HT), and 2 μΜ _DL_-propargylglycine (PAG), alone or their combinations. After 12 h, the confocal images of AzMC-dependent and DCF-dependent fluorescence in seedling roots were used to represent endogenous H_2_S (**a**) and H_2_O_2_ (**b**) contents. Scale bar = 200 μm. Meanwhile, the relative fluorescence presented as values relative to Con (**c**, **d**). Also, the emerged LR density and the number of emerged LRs (>1 mm) per seedling (**e**) were analyzed with plants 4 d after treatments. Mean and SE values were calculated from at least three independent experiments. Within each set of experiments, bars denoted by the same letter did not differ significantly at *P* < 0.05 according to Duncan’s multiple range test

Compared with NaHS alone, the decreased H_2_S production (determined by spectrophotography) and thereafter the impaired LR formation were previously observed when hypotaurine (HT; a H_2_S scavenger) was added together with NaHS [23]. To further confirm whether above NaHS response was H_2_S-dependent, a commercial specific fluorescent probe AzMC for H_2_S was applied. As expected, when together with HT or _DL_-propargylglycine (PAG; a synthetic inhibitor of H_2_S), AzMC-related florescent density and LR formation achieved by NaHS were impaired as well (Fig. 1a–e). Above results clearly confirmed that the response of NaHS in the induction of LR formation was H_2_S-dependent.

**Fig. 2:**
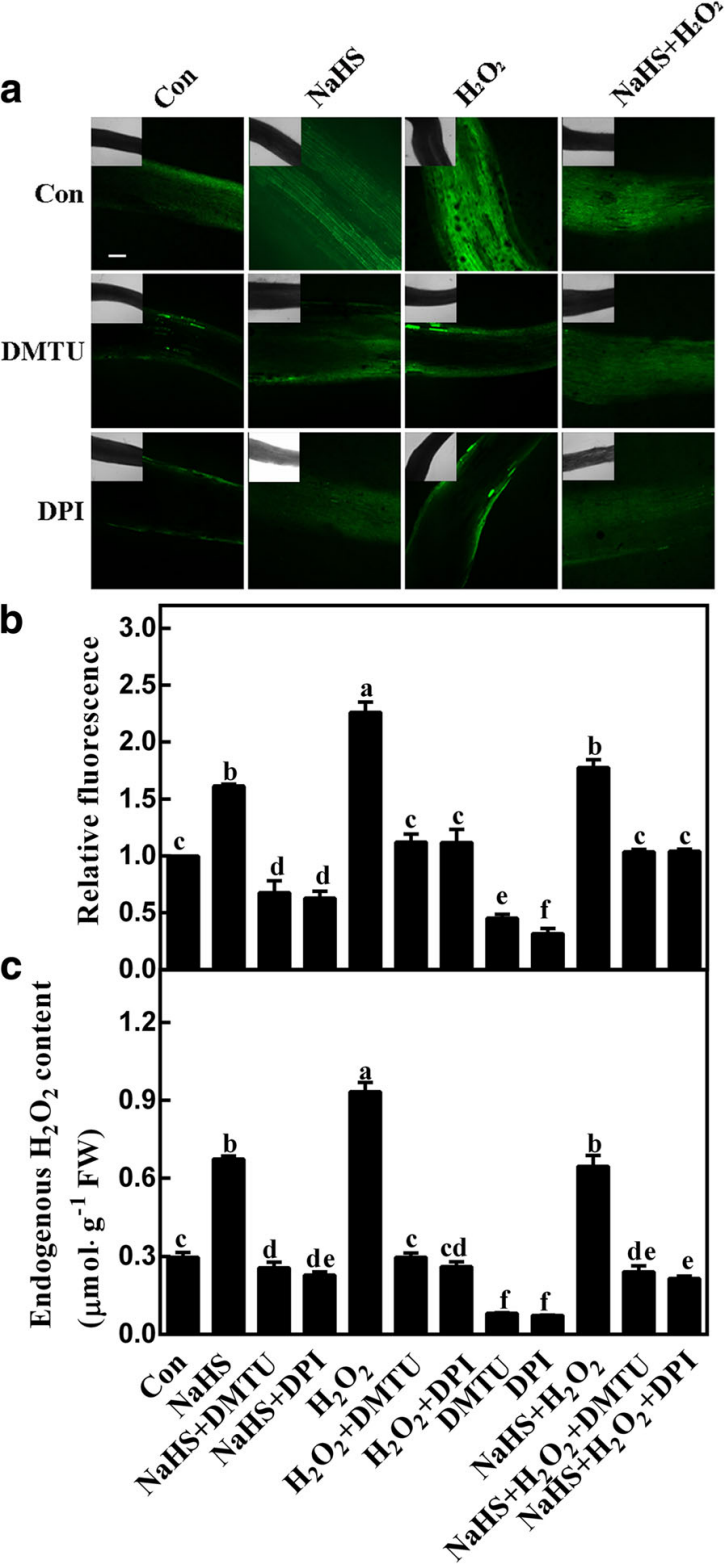
H_2_S-induced H_2_O_2_ accumulation is diminished by the scavenger and synthetic inhibitor of H_2_O_2_. Three-day-old tomato seedlings were treated with H_2_O (Con), 1 mM NaHS, 100 μΜ H_2_O_2_, 500 μΜ *N,N′*-dimethylthiourea (DMTU), and 0.1 μΜ diphenylene idonium (DPI), alone or their combinations for 12 h. Afterwards, corresponding confocal images of DCF-dependent fluorescence in seedling roots were provided to represent endogenous H_2_O_2_ contents (**a**), and the relative fluorescence were presented as values relative to Con (**b**). Scale bar = 200 μm. Meanwhile, the H_2_O_2_ contents were determined by spectrophotography (**c**). Mean and SE values were calculated from at least three independent experiments. Bars with different letters denoted significant differences at *P* < 0.05 according to Duncan’s multiple range test

Further, seedlings were loaded with reactive oxygen species (ROS)-specific fluorescent dye H_2_DCF-DA, and laser confocal scanning microscopy (LCSM) was used to investigate changes in ROS-induced fluorescence. Meanwhile, exogenously applied with H_2_O_2_ was regarded as a positive control. Figure 2a and b showed the images and quantified the fluorescence levels detected in H_2_O_2_-treated seedlings in the presence or absence of DMTU (a H_2_O_2_ scavenger) or DPI (an inhibitor of NADPH oxidase). Results revealed that both DMTU and DPI reduced, at least partially, the DCF-dependent fluorescence in the root tissues, consistent with the explanation that some, if not most, of the fluorescence was caused by endogenous H_2_O_2_. Thus, the fluorescence was used to report endogenous H_2_O_2_ levels throughout this study.

Subsequent results revealed that endogenous H_2_O_2_ production was induced as well when NaHS was applied, since the DCF-dependent fluorescence was increased by 56%, compared to the control samples (Fig. 1b, d). By contrast, the addition of HT and PAG weaken above fluorescence induced by NaHS, suggesting that NaHS-induced H_2_O_2_ might be obviously blocked by the removal of H_2_S. Meanwhile, HT or PAG alone, not only decreased corresponding fluorescence, but also inhibited LR formation (Fig. 1e). Combined with the changes in LR density and its number, we thus speculated a potential interrelationship between endogenous H_2_S and H_2_O_2_ during lateral root formation.

### **H**_**2**_**S**-induced tomato lateral rooting is sensitive to the removal of **H**_**2**_**O**_**2**_

**Fig. 3:**
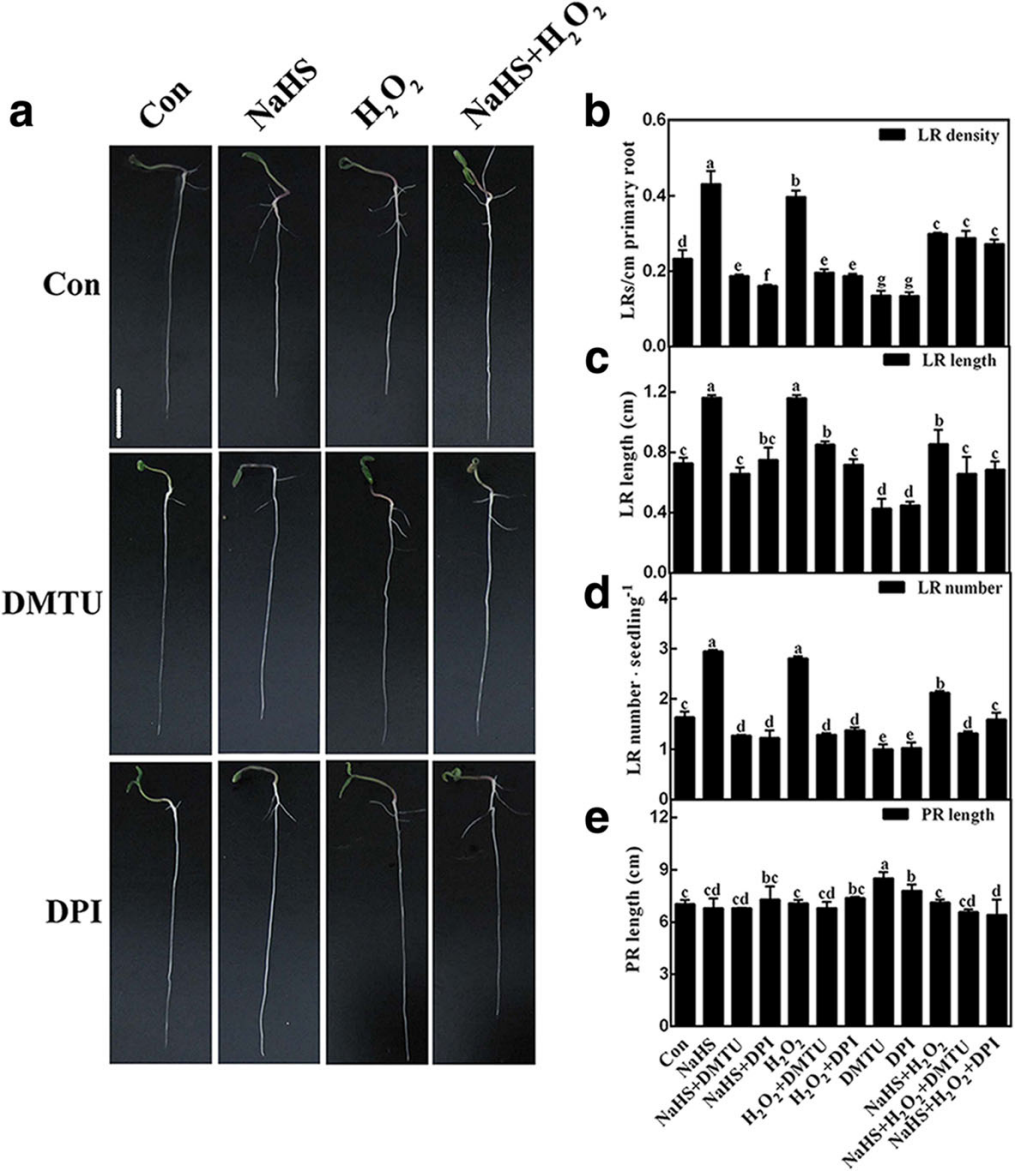
H_2_S-induced tomato lateral rooting is sensitive to the scavenger and synthetic inhibitor of H_2_O_2_. Three-day-old tomato seedlings were treated with H_2_O (Con), 1 mM NaHS, 100 μΜ H_2_O_2_, 500 μΜ *N,N′*-dimethylthiourea (DMTU), and 0.1 μΜ diphenylene idonium (DPI), alone or their combinations for 4 d. Corresponding photographs were taken (**a**). Bar = 1 cm. Meanwhile, the emerged LR density (**b**), LR length (**c**), the number of emerged LRs (>1 mm) per seedling (**d**), and primary root (PR) length (**e**) were analyzed. Mean and SE values were calculated from at least three independent experiments. Bars denoted by the same letter did not differ significantly at *P* < 0.05 according to Duncan’s multiple range test

**Fig. 4:**
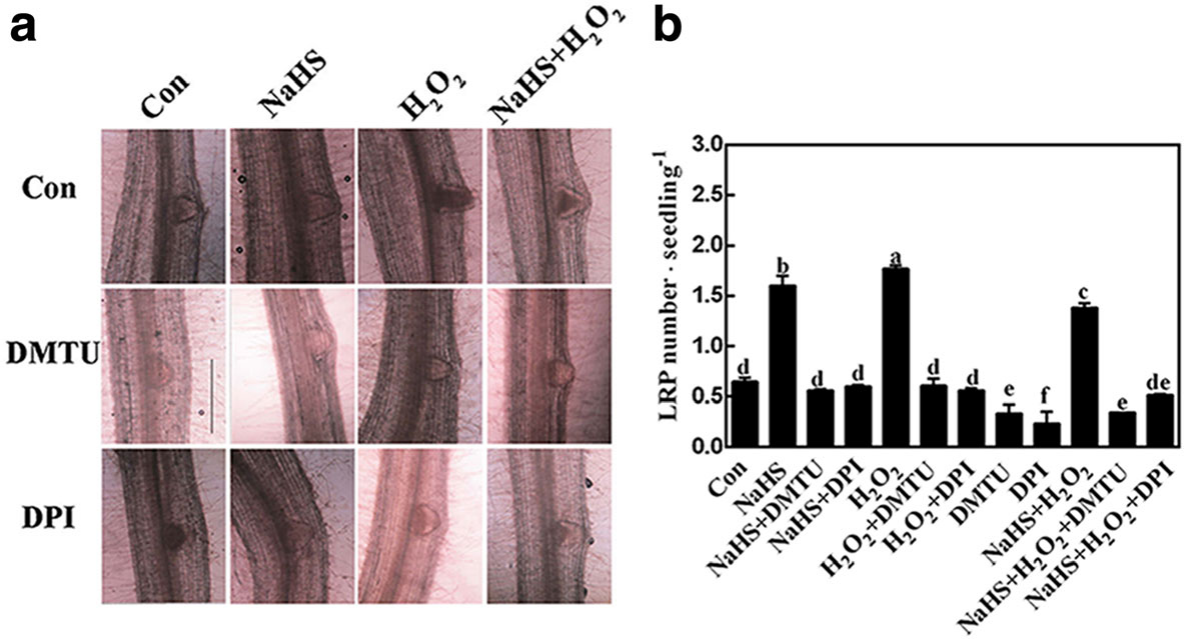
H_2_S-induced lateral root primordial (LRP) formation is sensitive to the removal of H_2_O_2_. Three-day-old tomato seedlings were treated with H_2_O (Con), 1 mM NaHS, 100 μΜ H_2_O_2_, 500 μΜ *N,N′*-dimethylthiourea (DMTU), and 0.1 μΜ diphenylene idonium (DPI), alone or their combinations. After various treatments for 3 d, photographs showing the representative morphology of LRP (about 75% of LRP at the shown stages), were taken (**a**). Bar = 0.25 mm. Meanwhile, the number of emerged LRP was also analyzed (**b**). Mean and SE values were calculated from at least three independent experiments. Bars with different letters denoted significant differences at *P* < 0.05 according to Duncan’s multiple range test

To investigate the contribution of H_2_O_2_ during LR formation triggered by H_2_S, DMTU and DPI were used together with NaHS and H_2_O_2_ to evaluate tomato LR development. The results shown in Fig. 3 indicated that the addition of DMTU or DPI alone could bring about decreases in LR density (Fig. 3a and b), LR length (Fig. 3c), and LR number (Fig. 3d); while, the primary root (PR) length was increased (Fig. 3e). Further experiment revealed that both NaHS- and H_2_O_2_-induced lateral rooting were greatly reduced in the presence of DMTU and/or DPI. Microscopical analysis showed that NaHS- and H_2_O_2_-induced LR primordia (LRP) presented a similar anatomic structure, and the inducing effects achieved by NaHS and H_2_O_2_ could be apparently prevented by DMTU or DPI (Fig. 4). Above results indicated a hypothesis that endogenous H_2_O_2_ might be required for H_2_S-induced lateral root development. Additionally, no additive responses were found when NaHS and H_2_O_2_ were applied together.

### **H**_**2**_**O**_**2**_ is required for lateral root formation triggered by **H**_**2**_**S**

The role of H_2_O_2_ in H_2_S-induced lateral root development was further examined by monitoring H_2_O_2_ synthesis in response to applied NaHS. As expected, a significant increase in H_2_O_2_-related fluorescence was observed in NaHS-treated tomato seedling roots compared with control sample (*P* < 0.05), suggesting H_2_S-mediated H_2_O_2_ production (Fig. 2a and b). This deduction was confirmed by the co-treatment with DMTU and DPI. We also noticed that when NaHS was together with H_2_O_2_, there is no additive response in the fluorescence. The changes of endogenous H_2_O_2_ detected with spectrophotography showed the similar tendencies (Fig. 2C).

**Fig. 5:**
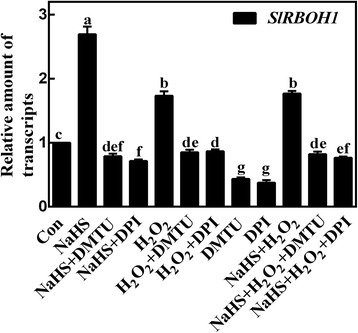
H_2_S modulates the expression of *SlRBOH1*. Three-day-old tomato seedlings were treated with H_2_O (Con), 1 mM NaHS, 100 μΜ H_2_O_2_, 500 μΜ *N,N′*-dimethylthiourea (DMTU), and 0.1 μΜ diphenylene idonium (DPI), alone or their combinations, for 6 h. Afterwards, the amount of transcript were analyzed by qPCR, and presented relative to the Con. Mean and SE values were calculated from at least three independent experiments. Bars with different letters denoted significant differences at *P* < 0.05 according to Duncan’s multiple range test

In order to assess the possible source(s) of H_2_O_2_, we thus evaluated the expression of *RBOH1*, the key gene responsible for H_2_O_2_ synthesis in tomato seedling roots [40]. As expected, a significant increase of *RBOH1* expression was observed when tomato seedlings were incubated with NaHS, and the up-regulation of *RBOH1* transcript was reversed by DMTU or DPI (Fig. 5). Meanwhile, a significant but weaker induction in *RBOH1* transcript was observed in response to the addition of H_2_O_2_ with or without NaHS. Above results indicated that H_2_O_2_ might be required for LR formation elicited by H_2_S.

### **H**_**2**_**O**_**2**_ modulates the expression of cell cycle regulatory genes in **H**_**2**_**S-**induced **LR** formation

**Fig. 6:**
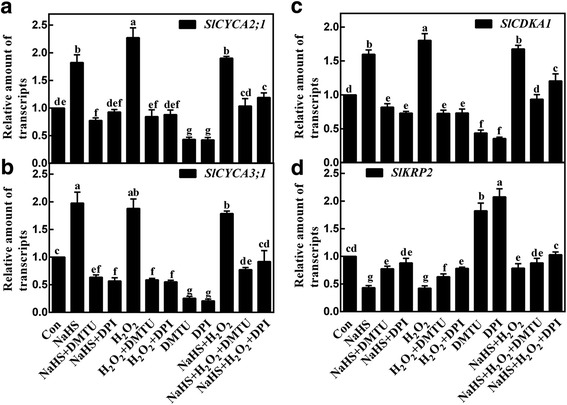
H_2_S affects the expression of *SlCYCA2;1*, *SlCYCA3;1*, *SlCDKA1*, and *SlKRP2*. Three-day-old tomato seedlings were treated with H_2_O (Con), 1 mM NaHS, 100 μΜ H_2_O_2_, 500 μΜ *N,N′*-dimethylthiourea (DMTU), and 0.1 μΜ diphenylene idonium (DPI), alone or their combinations for 12 h. Afterwards, *SlCYCA2;1* (**a**), *SlCYCA3;1* (**b**), *SlCDKA1* (**c**), and *SlKRP2* (**d**) transcript levels were analyzed by qPCR, and presented relative to the Con. Mean and SE values were calculated from at least three independent experiments. Bars with different letters denoted significant differences at *P* < 0.05 according to Duncan’s multiple range test

To further study the potential relationship between H_2_O_2_ and H_2_S in the induction of LR formation, the influence of NaHS, H_2_O_2_, DMTU, and DPI applied alone or their combination on the expression of cell cycle regulatory genes, was analyzed by qPCR. Similar to the inducible effects triggered by H_2_O_2_, NaHS resulted in the up-regulation of *SlCYCA2;1*, *SlCYCA3;1*, and *SlCDKA1* transcripts, together with simultaneous down-regulation of *SlKRP2* transcripts (Fig. 6). However, DMTU or DPI significantly blocked above mentioned modulation in these transcripts triggered by treatments with NaHS and/or H_2_O_2_. These results indicated that H_2_S-triggered LR formation was likely to be achieved by up-regulation of H_2_O_2_-mediated cycle regulatory genes.

### Expression of miRNAs and their target genes

**Fig. 7:**
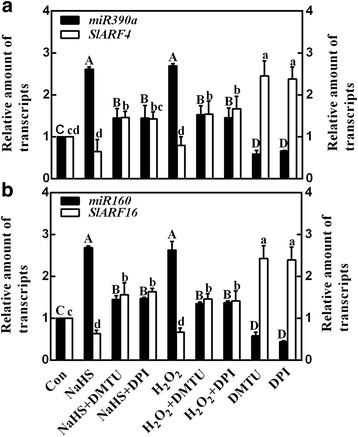
H_2_S affects the expression of microRNAs and their target genes. Three-day-old tomato seedlings were treated with H_2_O (Con), 1 mM NaHS, 100 μΜ H_2_O_2_, 500 μΜ *N,N′*-dimethylthiourea (DMTU), and 0.1 μΜ diphenylene idonium (DPI), alone or their combinations for 12 h. Meanwhile, *miR390a* (**a**; black), *SlARF4* (**a**; white), *miR160* (**b**; black), and *SlARF16* (**b**; white) transcript levels were analyzed by qPCR, and presented relative to the Con. Mean and SE values were calculated from at least three independent experiments. Within each set of experiments, bars with different letters denoted significant differences at *P* < 0.05 according to Duncan’s multiple range test

In the subsequent experiments, several LR formation-related miRNAs and their target genes were investigated to check whether they were involved in H_2_S-triggered LR development. Results shown in Fig. 7 revealed that both NaHS and H_2_O_2_ up-regulated *miR390a* and *miR160* transcripts; while, their corresponding target genes, including *SlARF4* and *SlARF16*, were significantly reduced. Contrasting changes were observed when NaHS or H_2_O_2_ was added together with DMTU or DPI. Above results confirmed the opposite effects between changes in miRNAs and their target genes.

### Detection of *S*-sulfhydrated proteins in H_2_S-treated tomato

**Fig. 8:**
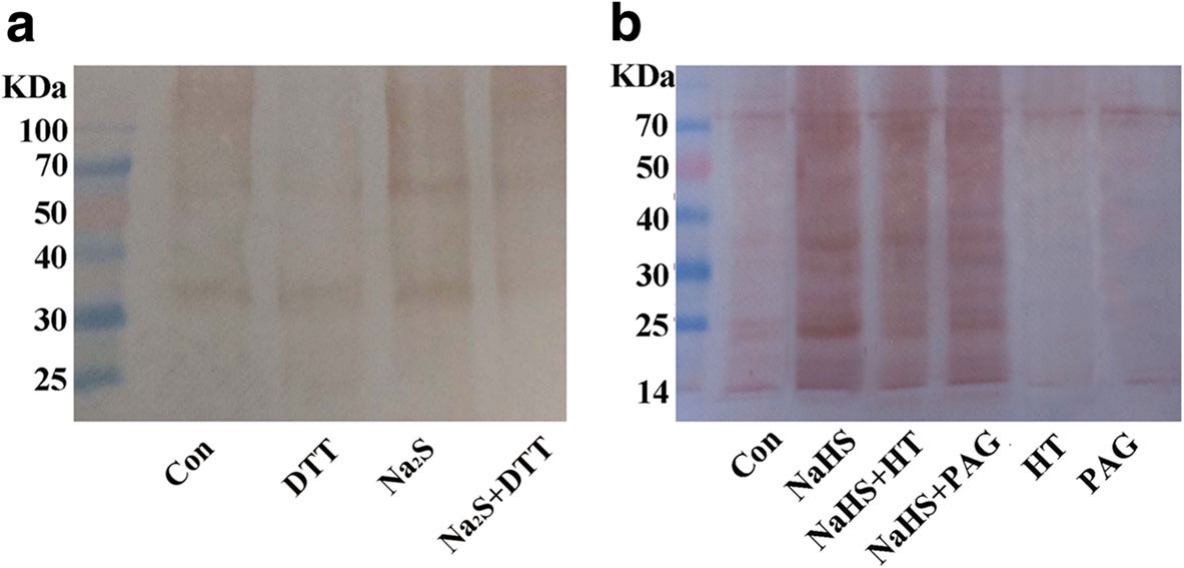
Detection of *S*-sulfhydrated proteins. **a** Protein extracts from 0.25 g of tomato roots were exogenously treated with H_2_O (Con), 2 mM Na_2_S (for 1 h; another H_2_S donor), and 2 mM DTT (for 30 min; a sulfhydration inhibitor), alone or their combinations (treatment with Na_2_S followed by DTT), and subjected to the modified biotin switch method (BSM). Finally, the labeled proteins were detected using protein blot analysis with antibodies against biotin. **b** Three-day-old tomato seedlings were treated with H_2_O (Con), 1 mM NaHS, 200 μΜ hypotaurine (HT), and 2 μΜ _DL_-propargylglycine (PAG), alone or their combinations for 4 d. Afterwards, protein extracts from 0.25 g of seedling roots were subjected to the BSM, and the labeled proteins were detected using protein blot analysis with antibodies against biotin. Representative pictures were provided

To further analyze the molecular mechanism underlying H_2_S signaling in LR formation, the pattern of *S*-sulfhydrated proteins in tomato roots was analyzed by using the modified biotin switch method. The results shown in Fig. 8a illustrated that treatment of tomato root extraction with Na_2_S (another H_2_S donor; [26]) enhanced *S*-sulfhydration, which was alleviated by DTT (a sulfhydration inhibitor; [24]). Consistently, tomato seedlings were treated with NaHS, HT, and PAG, alone or their combinations, then root extracts were used to analysis *S*-sulfhydrated profiles (Fig. 8B). Similarly, NaHS increased the level of *S*-sulfhydrated proteins, which was partially blocked by HT or PAG. Additionally, in compared with the control samples, HT or PAG alone slightly decreased sulfhydration.

## Discussion

H_2_S is proposed as the third gas messenger after NO and CO to fulfill many important roles in plants, including the inducement of LR formation [23, 41, 42]. The important function of H_2_O_2_ in the auxin-induced LR formation was also illustrated [39]. Although H_2_O_2_ involved in H_2_S-induced salt tolerance pathway of the Arabidopsis root was discovered [43], the relationship between H_2_S and H_2_O_2_ in LR formation is largely unclear. Here, we provided evidence for a previously unknown role for H_2_O_2_ in H_2_S-triggered LR formation in tomato seedlings.

Firstly, our results showed that an increase in the concentration of endogenous H_2_O_2_ determined by spectrophotography and LSCM, is one of the earliest responses involved in the signaling pathway governing LR formation triggered by H_2_S (Figs. 1 and 2). These results are in agreement with those obtained in Arabidopsis subjected to salinity stress [43], showing that NaHS induced a gradual elevation of H_2_O_2_ in NaCl-stressed seedling roots. This is an important point, since H_2_O_2_ is regarded as one of the ubiquitous components of the signaling transduction pathway [29], including responsible for the induction of LR formation [38, 39, 44] and adventitious rooting [34, 35].

Further pharmacological and microscopical evidence revealed the requirement of endogenous H_2_O_2_ in the induction of tomato LR formation triggered by H_2_S. This conclusion is based on several pieces of evidence: (i) the removal of endogenous H_2_O_2_ by its membrane-permeable scavenger DMTU impaired the induction of LR formation elicited by H_2_S (Figs. 2 and 3); (ii) the similar inhibiting responses triggered by DPI, an inhibitor of NADPH oxidase, in H_2_S-induced H_2_O_2_ production (Fig. 2) and thereafter LRP formation and lateral rooting (Figs. 3 and 4) were significant, implying the involvement of tomato RBOH1, at least partially. Changes in *SlRBOH1* transcripts confirmed this deduction (Fig. 5). Certainly, other candidate(s) for H_2_O_2_ synthesis (such as PAO and DAO; [36]) could not be easily ruled out in this process. Although we can not exclude the possibility that above mentioned chemicals may not specifically target H_2_O_2_, above results clearly indicated that H_2_O_2_ might be the downstream messenger of H_2_S signaling responsible for LR formation. This deduction was consistent with the recent genetic results [45], showing that *RBOH*-mediated ROS production facilitated LR emergence in Arabidopsis.

Strong evidence proved that the expression of cell cycle regulatory genes plays important roles in the early LR initiation in the presence of auxin and NO [3, 4, 6]. Similar to the previous results [23], our further molecular evidence revealed that H_2_S could modulate four cell cycle regulatory genes, including *SlCYCA2;1*, *SlCYCA3;1 SlCDKA1* and *SlKRP2*, mimicking the actions of H_2_O_2_ (Fig. 6). By contrast, the blocking effects were observed when DMTU or DPI was respectively supplemented together with H_2_S and/or H_2_O_2_. Combined with the changes in phenotypes (Fig. 3), we further speculated that H_2_S-triggered H_2_O_2_ was important in the early LR initiation by targeting cell cycle regulatory genes.

It is well-known that plant miRNAs play an important role in leaf morphogenesis [46], leaf polarity [47, 48], flowering time [49, 50], and flower development [51]. Some studies also focused on miRNAs related to plant root organogenesis [52]. For example, Marin et al. [13] and Yoon et al. [53] revealed that *miR390* and AUXIN RESPONSE FACTORS (ARFs) formed an auxin-responsive regulatory network (*miR390-TAS3*-*ARF2/ARF3/ARF4*) controlling lateral root development. Another miRNA, *miR160*, was confirmed to have a positive role in the induction of LR formation via targeting *ARF16* in Arabidopsis [54]. Thus, several representative miRNAs correlated with ARFs and LR formation [52], including *miR390a* for *SlARF4* [55], and *miR160* for *SlARF16* [54], were chosen. In this study, the results of qPCR revealed that *miR390a* and *miR160* transcripts were increased by both H_2_S and H_2_O_2_, and contrasting changes were observed in their target genes, including *SlARF4* and *SlARF16* (Fig. 7). Above mentioned changes were obviously prevented by the removal of endogenous H_2_O_2_ when DMTU or DPI was added together. These results were consistent with the changes in endogenous H_2_O_2_ levels (Fig. 2) and thereafter LR formation (Fig. 3). Thus, we deduced that auxin signaling mediated by H_2_O_2_-elicited miRNAs expression might be the important mechanism responsible for LR formation triggered by H_2_S. Certainly, corresponding genetic evidence should be investigated in the near future.

Recently, H_2_S-dependent *S*-sulfhydration, the conversion of cysteine -SH residues to persulfide (−SSH) which could be detected by using a modified biotin switch method, has been described to play a vital role in mammalians and plants [26, 27]. Nevertheless, whether *S*-sulfhydration was involved in plant LR formation is still unknown. In our experimental conditions, the *S*-sulfhydration conditions were strengthened when protein extracts from tomato seedling roots were treated with Na_2_S (another H_2_S donor; [26]), and the addition of DTT (a sulfhydration inhibitor; [24]) impaired above effect (Fig. 8a). Since DTT could reduce disulfide bonds, our results suggested that the modification is covalent and involves a sulfhydryl group. Similar results were obtained when tomato seedlings were subjected to the chemicals related to the alternation of endogenous H_2_S homeostasis (Fig. 8b). Thus, combine with the corresponding phenotypes in LR formation (Fig. 1), we provided a preliminary finding, that *S*-sulfhydration might be involved in H_2_S*-*promoted LR formation, although the specific *S*-sulfhydrated protein(s) had not been purified and elucidated. In fact, Aroca et al. [26] identified a total of 106 *S*-sulfhydrated proteins in *Arabidopsis*, and some of the proteins (ascorbate peroxidase and catalase; etc) identified were related to reactive oxygen species (ROS) metabolism. Since it was shown that ROS acted downstream of auxin action in the development of LR emergence [45], and ascorbate peroxidase (APX; a scavenging enzyme of H_2_O_2_) was previously confirmed to be *S*-sulfhydrated [26], the genetic and biochemical (in vitro and in vivo tests) approaches combined with proteomic and transcriptomic analyses should be applied to check whether APX acts as the *S*-sulfhydrated target of H_2_S signaling related to LR formation.

## Conclusions

**Fig. 9:**
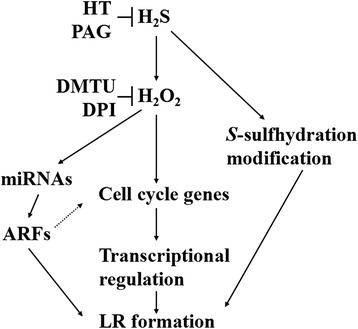
Schematic representation of the proposed model involving H_2_O_2_ homeostasis during H_2_S-triggered LR formation. The above pathway might be mediated by the expression of cell cycle genes in tomato seedlings. The involvement of miRNAs expression and *S*-sulfhydration modification were also suggested by solid lines. The possibility was suggested by dashed lines. T bars, inhibition

In summary, the results of this investigation indicated that an increase in H_2_O_2_ production might be an early response of H_2_S that contributes to the induction of LR formation by (i) modulating the expression of cell cycle regulatory genes; (ii) regulating auxin signaling mediated by miRNAs expression; and (iii) at least partially involving *S*-sulfhydrated proteins (Fig. 9). Additionally, our results provide indications of transcriptional and post-translational regulatory mechanism that contributed to the development of LR formation elicited by H_2_S.

## Methods

### Chemicals

All chemicals were purchased from Sigma (St Louis, MO, USA) unless stated otherwise. Sodium hydrosulfide (NaHS) was used at 1 mM as a H_2_S donor. 200 μM hypotaurine (HT; an H_2_S scavenger) and 2 μM _DL_-propargylglycine (PAG; a synthetic inhibitor of H_2_S) were also used. Hydrogen peroxide (H_2_O_2_) was applied at 100 μM. *N,N′*-dimethylthiourea (DMTU), a scavenger of H_2_O_2_, was used at 500 μM. 0.1 μM diphenylene idonium (DPI) was regarded as an inhibitor of H_2_O_2_ synthetic enzyme (NADPH oxidase). A H_2_S fluorescent probe 7-azido-4-methylcoumarin (AzMC) and a reactive oxygen species (ROS) fluorescent probe 2′,7′-dichlorofluorescein diacetate (H_2_DCF-DA) were both used at a final concentration of 20 μM. According to our pilot experiments, the concentration of above chemicals exhibiting the effective responses was chosen.

### Plant material and growth conditions

Tomato (*Solanum lycopersicum* L.) seeds “baiguoqiangfeng” were surface-sterilized in 2% sodium hypochlorite for 6 min, rinsed extensively and germinated in distilled water at 25 ± 1 °C in the dark for 3 days. Afterwards, the selected identical seedlings with radicles 2–3 mm were transferred to 4 ml treatment solutions containing the indicated chemicals and grown in an illuminating incubator (25 ± 1 °C) with a light intensity of 200 μmol m^−2^ s^−1^ at 14/10 h (light/dark) photoperiod.

After treatments for 4 d or the indicated time points, photographs were taken. Meanwhile, according to the previous methods [23, 39], the number of emerged lateral roots (LRs; >1 mm) per seedling, the length of primary root (PR), the length of LR and the emerged LR density (the number of LR per cm primary root; LRs/cm) were determined by using Image J software. Additionally, LR primordial (LRP) per seedling were observed after 3 d of treatments by root squash preparations and quantified with a light microscope. Unless stated otherwise, only the lateral root-inducible segments were used for the subsequent biochemical and molecular analyses. Thus, the root apical meristems were cut off, and the shoots of seedlings were removed by cutting below the root-shoot junction.

### Laser scanning confocal microscopy (LSCM)

According to the previous methods with minor modification [56, 57], endogenous H_2_O_2_ and H_2_S production were determined by a laser scanning confocal microscope (LSCM) using the ROS fluorescent probe H_2_DCF-DA and a H_2_S fluorescent probe AzMC. After treatments, roots were incubated in 20 mM HEPES-NaOH buffer (pH 7.5) containing 20 μM probe for 30 min in dark (25 °C). Afterwards, the roots were washed three times (15 min each time) with fresh HEPES buffer, and observed by using Zeiss LSM 710 confocal microscope (Carl Zeiss, Oberkochen, Germany) with the same exposure time.

All manipulations were performed at 25 °C. Each photograph were taken at the eyepiece 5 × magnification based on 20 overlapping confocal planes of 15 μm each using ZEN software (300 μm sections along Z stack). For each picture, the overall fluorescence of maturation zone of the primary root (about an area of 500,000 μm^2^), where cells become differentiated, and at a later stage lateral roots emerge, was quantified [9]. The bright-field (BF) images corresponding to the fluorescent images were also shown at the top left corners of the photograph. Representative photographs with similar results were obtained after the analysis of at least fifteen samples for each experiment. Afterwards, the average intensities of 15 photographs (1 photograph per sample) for each treatment were calculated. The relative fluorescence was presented as values relative to control sample.

### Measurement of **H**_**2**_**O**_**2**_ content

The content of H_2_O_2_ was analyzed by the FOX1 method [58, 59]. Samples were extracted with 200 mM perchloric acid (HClO_4_). After centrifugation at 4 °C, 10,000 *g* for 15 min, 500 μL supernatant was transferred to 500 μL assay solution containing 500 μM ammonium ferrous sulfate, 50 mM H_2_SO_4_, 200 μM xylenol orange, and 200 mM sorbitol, for 45 min in dark (25 °C). Afterwards, the absorbance values were detected at 560 nm. The specificity for H_2_O_2_ was tested by eliminating H_2_O_2_ in the reaction mixture with catalase (CAT). Standard curves of H_2_O_2_ were obtained for each independent experiment by adding variable amounts of H_2_O_2_.

### Quantitative real-time RT-PCR (qPCR) analysis

qPCR was used to analyze the expression of cell cycle regulatory genes, ARFs genes, and miRNA. After various treatments, total RNA from about 100 mg (fresh weight) samples was isolated by using Trizol reagent (Invitrogen, Gaithersburg, MD, USA). Afterwards, the RNA samples were reverse-transcribed using an oligo d(T) primer and M-MLV reverse transcriptase (BioTeke, Beijing, China). Quantitative RT-PCR reactions were performed using a Mastercycler® ep *realplex* real-time PCR system (Eppendorf, Hamburg, Germany) with SYBR® *Premix Ex Taq*™ (TransGen Biotech, Beijing, China) according to the manufacturer’s instructions. The accession numbers (GenBank/miRBase) and oligonucleotide primers were shown in Additional file 1: Table S1. Three biological and three technological repeats were performed in qPCR. Relative expression levels of corresponding genes were calculated by using the 2^−ΔΔ*C*^_T_ method [60, 61], and were presented as values relative to that of corresponding control samples at the indicated times, after normalization with *Actin* and *GAPDH* transcript levels.

A One Step PrimeScript miRNA cDNA synthesis kit (TaKaRa Bio Inc., Dalian, China) was used to synthesize cDNA for analyzing miRNA expression by qPCR. The specific 5′ primers were listed in Additional file 1: Table S1. The 3′ primer was supplied in the kit. *U6 snRNA* was used as internal control. The rest steps were the same as the approaches described previously [62].

### Modified biotin switch method

The modified biotin switch method was carried out as previously described protocol with minor modification [26, 63]. Total proteins extracted from samples were homogenized in HEN buffer containing 250 mM Hepes-NaOH (pH 7.7), 1 mM EDTA and 0.1 mM neocuproine, and centrifuged at 10000 *g* for 15 min at 4 °C. The supernatant was transferred to fresh tubes, and added with three volumes of blocking buffer (HEN buffer supplemented with 2.5% SDS and 20 mM methyl methanethiosulfonate (MMTS)). Then, the solution was incubated at 4 °C for 12 h to block free sulfhydryl groups. The MMTS was then removed, and ice-cold acetone was used to precipitate the proteins at −20 °C for 20 min. After the removal of acetone, the proteins were resuspended in HENS buffer (HEN buffer supplemented with 1% SDS). Afterwards, the *S*-sulfhydrated proteins were labeled using 4 mM *N*-[6-(biotinamido)hexyl]-3′-(2′-pyridyldithio)propionamide (Biotin-HPDP) for 3 h at 25 °C in the dark.

The above biotin-labeled proteins were separated using non-reducing SDS-PAGE on 12% polyacrylamide gels. Then, the proteins were transferred to polyvinylidene fluoride membranes (Roche, Basel, Switzerland) according to the manufacturer’s instructions. Anti-biotin antibody (HRP) (Abcam antibodies, Cambridge, UK) was diluted 1:10,000. Meanwhile, Coomassie Brilliant Blue-stained gels were used to confirm the equal amounts of proteins loaded (data not shown).

### Statistical analysis

All results were shown as the mean values ± SE of at least three independent experiments with at least three biological replicates for each. By using SPSS 17.0 software, data was analyzed by one-way analysis of variance (ANOVA) followed by Duncan’s multiple range test, and *P* values <0.05 were considered statistically significant.

## Additional file

Additional file 1: Table S1. The accession numbers and primer sequences of real-time RT-PCR (qPCR). (DOC 45 kb)

## Abbreviations

3-MST: 3-Mercaptopyruvate sulfurtransferase
APX: Ascorbate peroxidase
ARFs: Auxin response factors
AzMC: 7-Azido-4-methylcoumarin
Biotin-HPDP: *N*-[6-(Biotinamido)hexyl]-3′-(2′-pyridyldithio)propionamide
CAT: Catalase
CBS: Cystathionine-*β*-synthase
CDK: Cyclin Dependent Kinases
CO: Carbon monoxide
CSE: Cystathionine-*γ*-lyase
DAO: Diamine oxidases
DMTU: Dimethylthiourea
DPI: Diphenylene idonium
H_2_DCF-DA: 2′,7′-Dchlorofluorescein diacetate
H_2_O_2_: Hydrogen peroxide
H_2_S: Hydrogen sulfide HRP Anti-biotin antibody
HT: Hypotaurine
LR: Lateral root
LRP: Lateral root primordial
LSCM: Laser scanning confocal microscopy
MiRNAs: MicroRNAs;
MMTS: Methyl methanethiosulfonate
NaHS: Sodium hydrosulfide
NO: Nitric oxide
PAG: _DL_-Propargylglycine
PAO: Polyamine oxidases
PR: Primary root
ROS: Reactive oxygen species
qPCR: Quantitative real-time RT-PCR
