# Supplementary material for: Hydrogen peroxide is involved in hydrogen sulfide-induced lateral root formation in tomato seedlings
Source: BMC Plant Biol. 2017 Oct 13;17:162. doi: 10.1186/s12870-017-1110-7 (PMC5640930; doi:10.1186/s12870-017-1110-7)
Supplement: Additional file 1: Table S1. — The accession numbers and primer sequences of real-time RT-PCR (qPCR). (DOC 45 kb) [file 12870_2017_1110_MOESM1_ESM.doc]

**Supplementary materials**

**Table S1. The accession numbers and primer sequences of real-time RT-PCR (qPCR)**

| Primer names | Accession number | Sequences (5’→3’) |
| --- | --- | --- |
| *Actin* | NM_001330119.1 | F: CCACGAGACTACATACAA |
|  |  | R: TACCACCACTGAGCACAA |
| *GAPDH* | NM_001247874.2 | F: AGGCTGGAATTGCTTTGAG |
|  |  | R: CCAGGCCCACAAAACTAA |
| *SlRBOH1* | NM_001247197.3 | F: AAGGAGTGGAGGGTGTGACT |
|  |  | R: AATGAGGCTCCGCCTAAACC |
| *SlCYCA2;1* | NM_001246839.2 | F: CATTAACAAGGGTATGCGAA |
|  |  | R: GTCAGGTAAAGAGTGTCCGG |
| *SlCYCA3;1* | NM_001247858.2 | F: TGCGGTTCTTGCCATCA |
|  |  | R: CGCCCAGTTGCTTCCA |
| *SlCDKA1* | NM_001247447.2 | F: CACTTGCCTGTCGCCTCCTC |
|  |  | R: ACCCCCTCGTCTTCCTGCTC |
| *SlKRP2* | NM_001247055.2 | F: CTTCACAAACCACCCACCCC |
|  |  | R: TTTCGTCCACCTCCCTCACC |
| *miR390a* | [MIMAT0035468](http://www.mirbase.org/cgi-bin/mature.pl?mature_acc=MIMAT0035467) | CGCTATCCATCCTGAGTTTTA |
| *SlARF4* | NM_001246842.1 | F: TTCGTCAGTTGCTACAGCCCTAT |
|  |  | R: TCGCTTATCCCAGTCACTACACC |
| *miR160* | [MIMAT0007914](http://www.mirbase.org/cgi-bin/mature.pl?mature_acc=MIMAT0007914) | TGCCTGGCTCCCTGTATGCCA |
| *SlARF16* | NM_001247951.2 | F: GATTTGTGACGGAAGACGAGAAC |
|  |  | R: ATCCCTGAGCACCATTGAACACT |
